# Lyme borreliosis diagnosis: state of the art of improvements and innovations

**DOI:** 10.1186/s12866-023-02935-5

**Published:** 2023-08-01

**Authors:** Mickaël Guérin, Marc Shawky, Ahed Zedan, Stéphane Octave, Bérangère Avalle, Irene Maffucci, Séverine Padiolleau-Lefèvre

**Affiliations:** 1 Unité de Génie Enzymatique Et Cellulaire (GEC), CNRS UMR 7025, Université de Technologie de Compiègne, 60203 Compiègne, France; 2 Connaissance Organisation Et Systèmes TECHniques (COSTECH), EA 2223, Université de Technologie de Compiègne, 60203 Compiègne, France; 3 Polyclinique Saint Côme, 7 Rue Jean Jacques Bernard, 60204 Compiègne, France

**Keywords:** Lyme disease, Tick, Diagnostic, Culture, PCR, Serology detection, Direct and indirect methods

## Abstract

With almost 700 000 estimated cases each year in the United States and Europe, Lyme borreliosis (LB), also called Lyme disease, is the most common tick-borne illness in the world. Transmitted by ticks of the genus *Ixodes* and caused by bacteria *Borrelia burgdorferi* sensu lato, LB occurs with various symptoms, such as erythema migrans, which is characteristic, whereas others involve blurred clinical features such as fatigue, headaches, arthralgia, and myalgia. The diagnosis of Lyme borreliosis, based on a standard two-tiered serology, is the subject of many debates and controversies, since it relies on an indirect approach which suffers from a low sensitivity depending on the stage of the disease. Above all, early detection of the disease raises some issues. Inappropriate diagnosis of Lyme borreliosis leads to therapeutic wandering, inducing potential chronic infection with a strong antibody response that fails to clear the infection. Early and proper detection of Lyme disease is essential to propose an adequate treatment to patients and avoid the persistence of the pathogen. This review presents the available tests, with an emphasis on the improvements of the current diagnosis, the innovative methods and ideas which, ultimately, will allow more precise detection of LB.

## Introduction

**Fig. 1:**
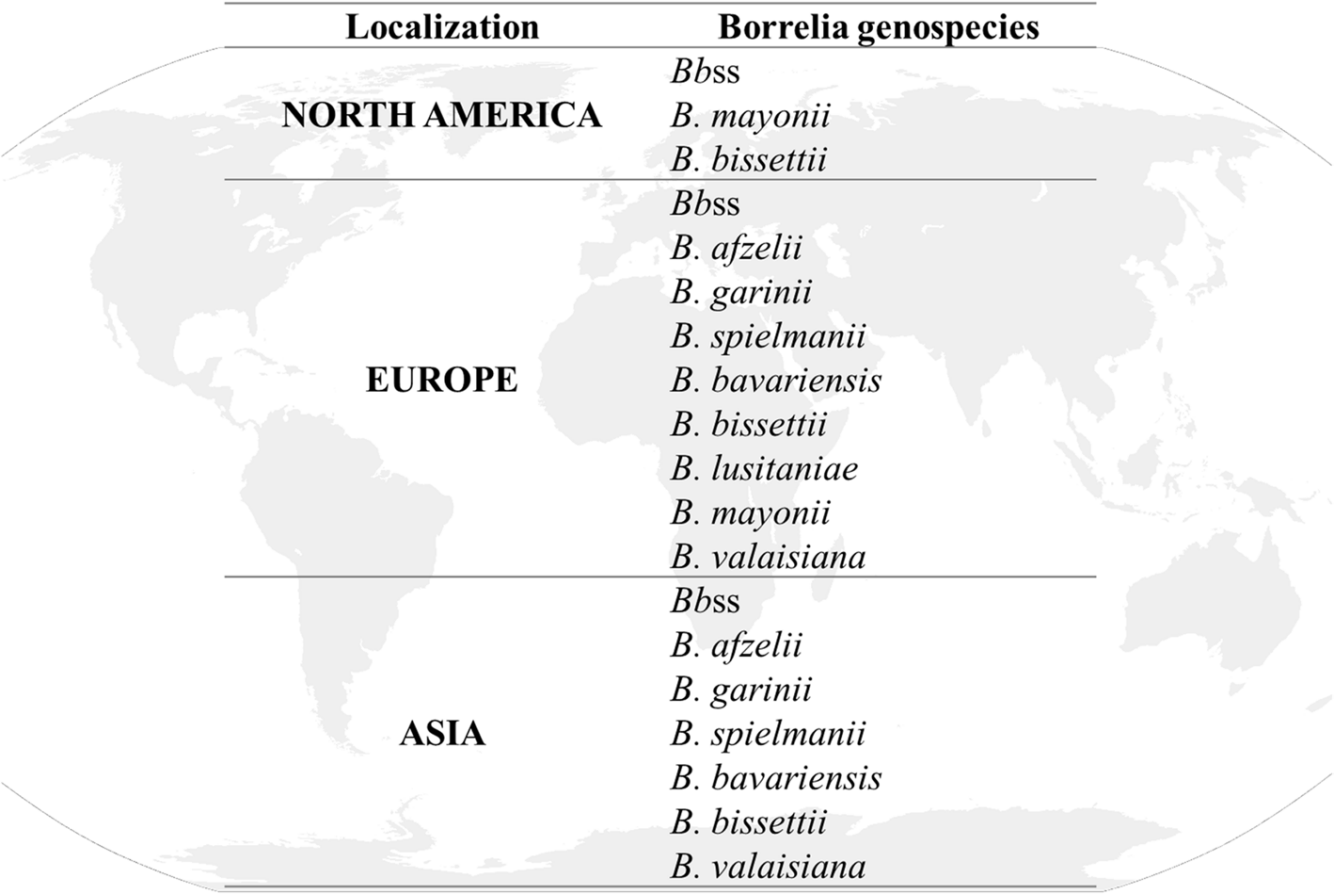
Geographic distribution of *Borrelia* species

Lyme borreliosis (LB), discovered in 1975 and commonly known as Lyme disease, is transmitted by ticks of the genus *Ixodes*. Etiological agents are the spirochete bacteria from the *Borrelia burgdorferi* sensu lato complex (*Bb*sl). The distribution of the various genospecies is detailed in Fig. 1 [1, 2]. Recent studies aim to update the genospecies distribution worldwide for better diagnosis of LB [3–6].

The genospecies, reported inside the *Borreliae* Lyme Group (*B*LG), are different in terms of clinical manifestation, dissemination insides the human body but also in terms of genomes/antigens expression. Regarding the symptoms, it has been described for example that, *Bb*ss has shown to be arthritogenic, whereas *B. afzelii* causes skin infections and *B. garinii* is especially neurotropic. Recently, *B. mayonni,* member of the *B*LG, has been associated with unusually high spirochaetemia, like Tick Borrelia Relapsing Fever (TBRF) genospecies, while the others genospecies causing LB are characterized by being organotrophic with a very low level of spirochaetemia [2, 7, 8].

The heterogeneity of both the clinical manifestation and the species distribution worldwide leads to regional differences in terms of diagnosis [9]. Indeed, when focusing on the regional genospecies differences and concerning the genome of the complex *Bb*sl, it has been shown that even if the linear chromosome of 910 kilobases is highly conserved among the *B*LG, the plasmids (9 linear plasmids and 12 circular plasmids) [10] show a high degree of variation [9, 11, 12]. Some researchers have shown, for example by studying OspC (Outer Surface Proteins) typing or by multilocus sequence analysis (MLSA), many differences between genospecies in Europe and the United States [13–15]. Intraspecies diversity has also been noted, in the US, where patients from New York and Wisconsin were infected by two distinct populations of *Bb*ss but genetically and phylogenetically closely related [16, 17]. The intricate nature of diverse Borrelia genospecies within Europe and in the US, added to the antigenic variation abilities of the genospecies, underscores the need for diagnostic assays capable of detecting all pathogenic strains.

LB is the most common tick-borne illness and every year affects for 476 000 estimated cases in the United States and more than 200 000 cases in Europe [18–20]. LB is underdiagnosed and/or misdiagnosed, making the estimated incidence controversial with probably a considerably higher number of cases [21].

Patients suffering from this disease have various symptoms depending on the stage of its evolution. Commonly divided into three stages—early localized, early disseminated, and late disseminated—the only pathognomonic symptom, erythema migrans (EM), is a cutaneous manifestation, appearing during the first stage, at the site of the tick bite. However, EM is not systematically detected and only non-characteristic symptoms linked to stages 2 and 3 (Lyme neuroborreliosis, carditis, or arthritis) can be expressed [1, 22]. It is important to emphasize that the risk of the appearance of other symptoms from LB (especially neurologic and articular manifestations) is significantly reduced after the recognition of EM and the rapid initiation of antibiotic treatment [23].

Indeed, if LB is detected correctly, timely and early enough, its treatment is simple and involves an antibiotic therapy, which allows solving 80 to 90% of *Borrelia* infection cases [24]. However, the Lyme disease diagnosis is not straightforward, because of the above-mentioned blurred symptoms and the limits of the approved tests. Indeed, although other tests exist, the diagnosis of LB is currently based on serology using two tests: an enzyme-linked immunosorbent assay (ELISA) and a Western Blot (WB). These serological tests are indirect diagnostic methods measuring the presence of anti-*Bb* antibodies (Abs) [25]. Unfortunately, these techniques have many limitations [26]. *Bb*sl is able to escape the immune system according to a large variety of mechanisms, inducing a reduced immune protection despite the activation of innate and adaptive immunity [27]. Nevertheless, even if secreted antibodies fail to efficiently protect individuals, they should be exploitable for serodetection and indirect diagnosis. But, in practice, serodetection lacks of sensitivity in the early stage of the disease for many reasons, including, possibly, an insufficient time interval between the infection and the detection test, also called “window period”, or the use of antibiotic treatment limiting the development of a strong antibody response (see Sect. 1.1). Added to the possible cross-reactivity of antibodies, due to common antigens with other diseases, or to the co-infection issues suggesting that targeting a specific antibody is not sufficient [28], indirect measurement causes false positive or false negative results. Finally, the indirect approach doesn’t allow to distinguish between a past but cleared infection, and an active one [29]. Besides hampering the LB diagnosis and healing, the question of long or chronic form of the disease frequently raises [30]. Although still unclear, some hypotheses on the etiology of the persistent symptoms have been done, involving autoimmunity, cross-reactivity, molecular mimicry, co-infections/co-transmission, or borrelial tolerance to antibiotics [24, 31–33]. Detecting *Bb*sl bacteria despite treatment could help to decide if the antibiotics-based therapy has to be prolonged or not.

**Fig. 2:**
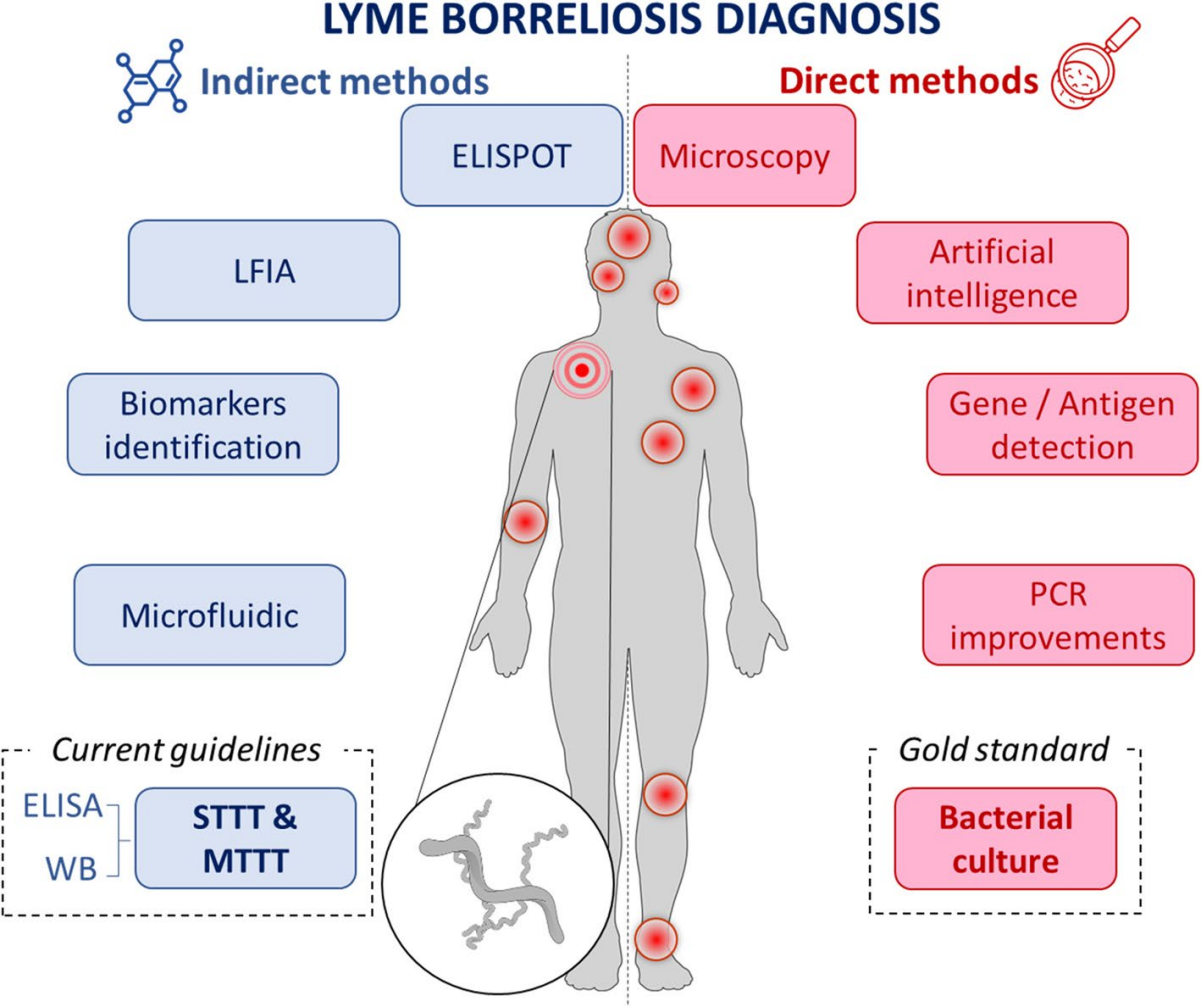
Holistic view of diagnostic approaches performed from patient fluids. Boxes in pink are direct detection methods whereas boxes in blue are indirect ones, potentially applied in the absence of Erythema migrans (represented by concentric circles). Others red spots represent some possible localizations of symptoms

In addition to previously published reports [18, 34–37], this review aims to update and wholly collect the knowledge and data available on the Lyme diagnosis issue, to summarize the limitations of direct and indirect LB detection methods, but also to present new strategies required to avoid therapeutic wandering (Fig. 2).

## Indirect diagnosis 

### Standard two-tiered testing (STTT) and modified two-tiered testing (MTTT)

Currently, serology testing, i.e. indirect diagnosis, is the official recommendation for the diagnosis of LB [38]. Serology is based on Abs detection following the immune response of the host after infection. However, serology encounters limitations to detect early localized Lyme disease, as well as early disseminated or late-stage LB. Indeed, due to the latency time of the immune response after bacterial or viral infection, detecting early Lyme disease, especially during its acute phase, using serology may be useless, since the diagnosis rarely exceeds a sensitivity beyond 50% [39]. Moreover, after treatment of an active infection, Abs can still be detected in serum from months to years after the infection [40]. This makes the serology unsuitable for monitoring response to treatment or identifying new infections.

Since 1994, the Centers for Disease Control and Prevention (CDC) guidelines and recommendations consist of a standard two-tiered serologic testing approach, called STTT, to maintain a high sensitivity and optimize the specificity. Firstly, an ELISA is performed. If the result is borderline or positive, a confirmatory test by WB is required [25].

**Fig. 3:**
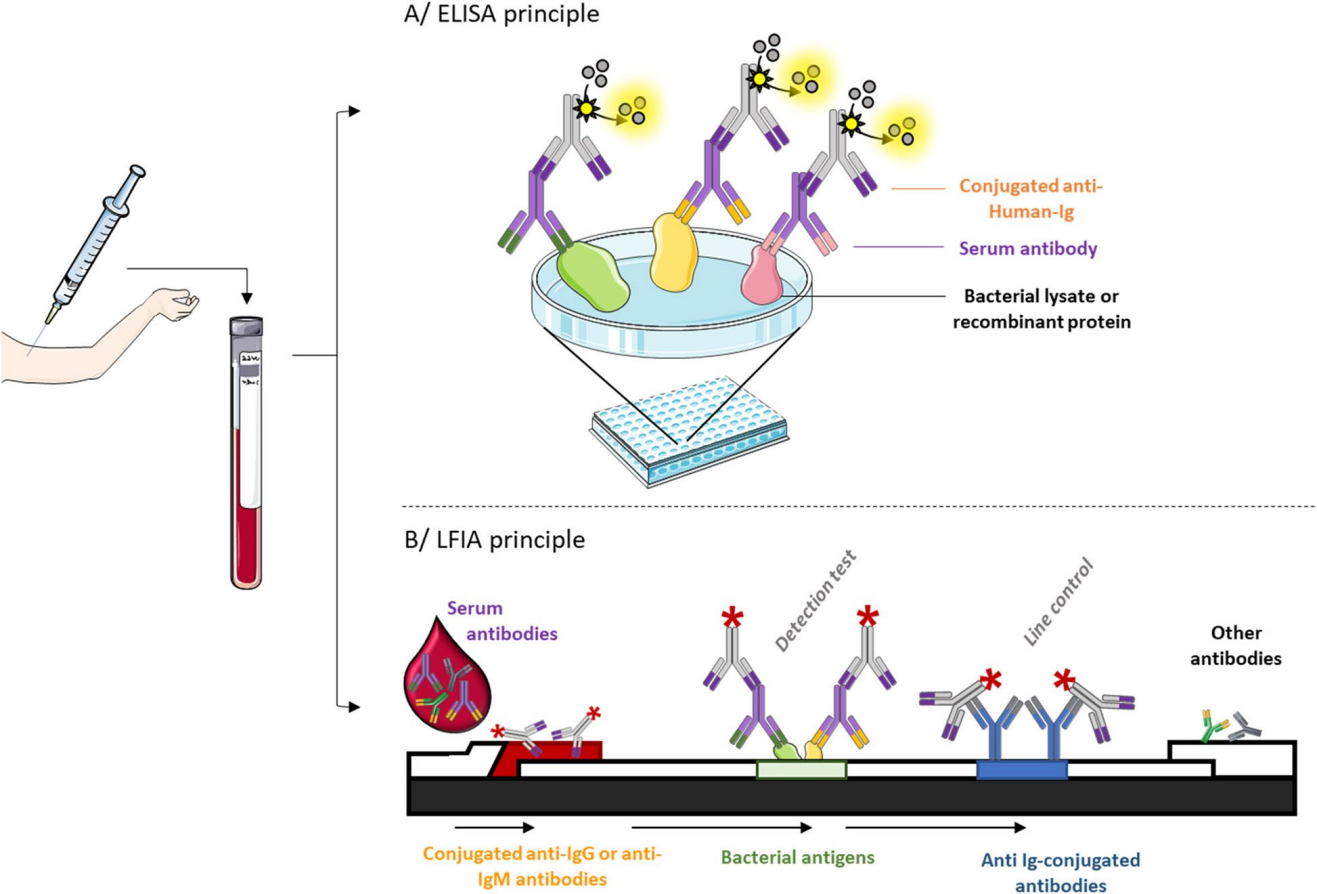
Indirect detection tests

The ELISA method is a very well-known technique used in diagnosis. It allows the detection of immunoglobulins (Ig) G and/or IgM. Briefly, antigens specific to *Bb* are coated on a plate and the patient’s sera are challenged for these antigens’ recognition. A secondary Ab labelled with an enzyme or a tag will bind to the crystallizable fragment (Fc) of the patient's Ab (Fig. 3A). Adding the substrate induces a color change or fluorescence reaction that can be measured with a suitable detection system. The reliability of ELISA depends on the antigen’s identity and its preparation. The first generation of ELISA for LB diagnosis was based on spirochete lysates obtained by sonication. However, this generation lacked sensitivity. Indeed, a large diversity of the antigen is thus coated on a plate, among which only a few are immunodominant. Thus, the ability to capture predominant antibodies is reduced. Furthermore, the expression of multiple antigens of *Bb* changes, depending of the bacteria’s localization (*in-vitro* culture, midgut of unfed ticks, when the tick starts feeding on mammals, or at different stages of human infection). The first generation of ELISA does not contain multiple antigens expressed later during the human infection such as VlsE (Variable major protein Like sequence Expressed) or OspC [41, 42]. This generation based on the Whole-Cell Sonicate (WCS) also lacked of specificity due to antibody cross-reactivity with proteins conserved between *Bb* and other commonly encountered bacteria (heat shock and flagellar proteins) [43]. Then, a second generation has been developed using purified, synthetic or recombinant antigens, such as the surface lipoproteins OspC, OspA, or VlsE [44]. Thus, the use of a synthetic peptide derived from the VlsE sequence (C6 peptide), which is highly immunogenic and well conserved [45, 46], or C10 peptide derived from the conserved amino-terminal portion of OspC (namely pepC10), is also used [47].

After a positive or borderline result, a WB is usually performed. The principle of WB is the same as ELISA. Abs produced consecutively to a *Bb* infection are eventually detected. The separation of *Bb* antigens by SDS-PAGE according to a characteristic migration profile preceding the detection confirms the data by increasing the specificity and by reinforcing the diagnosis. WB usually relies on *Bb* cell lysates and/or recombinant antigens [48, 49].

Sensitivity and specificity remain major issues for these techniques (Table 1). Besides the risk of false negative results due to poor sensitivity for early Lyme disease, a recent study showed that current tests generate also many false positives, i.e. showed poor specificity, leading to incorrect treatment of the patient [50]. In this work, the authors evaluated the reactivity of sera from patients with viral infections (Epstein-Barr virus (EBV) or cytomegalovirus (CMV)) with the *Borrelia* antigens used in the serological tests. Many false positives have been observed and are probably related to the cross-reaction of Abs produced during the lymphocyte response activated by the presence of viral superantigens. False positive enzyme immunoassay (EIA) is also reported due to the presence of cross-reactive Abs, due to common antigens with other diseases, including, but not only, tick-borne relapsing fever, anaplasmosis, and diseases related to the presence of *Helicobacter pylori* [51], *Treponema denticola* [38] and *T. pallidum,* the etiological agent of syphilis [52]. Much effort is made to overcome this problem. Recently, Arnaboldi et al. generated peptides from linear B cell epitope mapping. The purpose is dual: i) solve the specificity issues thanks to peptides—rather than complex protein mixes—able to specifically capture serum Abs, and ii) solve the sensitivity issues thanks to the use of a rationalized variety of such peptides, each representing an immunodominant epitope from a bacterial mix. The final aim is to ensure the development of a multi-peptide-based assay [53].

**Table 1:** Sensitivity and specificity for STTT in America and in Europe

Symptoms / Stage of the disease	Patient's localization					
	**America**			**Europe**		
	**Sensitivity**^**a**^	**Specificity**^**b**^	**References**	**Sensitivity**	**Specificity**	**References**
Erythema migrans or early Lyme borreliosis *Acute phase*^*c*^* (AP)*	11–50%	99%	[29, 34, 54–57]	23–55%	75–99%	[34, 58–61]
Erythema migrans or early Lyme borreliosis *Convalescent phase*^*d*^* (CP)*	18–78%	99%		30–67%	95%	[61]
Acute-phase neurologic or cardiac involvement	60–100%	99%		69–97%	98–99%	[58, 61]
Arthritis or late neurologic involvement	97–100%	99%		78–100%	98–99%	

^a^Ability of the test to identify the presence of a disease correctly (CDC)

^b^Ability of the test to identify the absence of a disease correctly (CDC)

^c^Phase during an active infection, characterized by active multiplication of the pathogens and pronounced symptoms

^d^Phase after the antibiotic treatment

Many reasons can also be singled out for influencing the STTT sensitivity and specificity, and should be nuanced. The first one is related to the studies themselves. Tests on patients with early LB have a lower sensitivity in the STTT and MTTT than patients with late LB. The main reason is the early LB patients do not have enough time to develop an effective antibody response. The second reason is depending of the patient’s treatment. Indeed, an effective antibiotic treatment during the early LB diminishes the antibody response from developing, meaning that patients are less likely to be tested positive on serological tests [62].

In contrast, patients with late LB such as Lyme arthritis, Lyme neuroborreliosis, or acrodermatitis chronica atrophicans (ACA) often have strong antibody responses to serological tests with high sensitivity and high specificity. Moreover, serological tests specificity is very dependent to the number of samples, especially between (i) healthy controls, (ii) potentially-cross reactional patients with others diseases, and (iii) LB patients. Finally, the patients’ localization used for the studies also greatly influences the sensitivity and the specificity. The main reasons are (i) the species involved in the disease (*Bb*ss in North America and *B. garinii, B. afzelii, B. spielmanii, B. bavariensis,* in Europe) and (ii) the criteria applied for considering the positivity of the diagnosis are different [58].

Besides the specificity and sensibility issues, STTT is time-consuming, requires skilled operators, suffers from subjective interpretation and from its inability to distinguish between an active and past infection [63]. These drawbacks highlight the need for improvements.

Since the introduction of the STTT at the Dearborn conference in 1994, variations in the combination of these tests have been proposed to improve the sensitivity and specificity [64, 65]. In July 2019, the Food and Drug Administration (FDA) cleared some EIAs to be used in a MTTT that might help to resolve the previously mentioned issues [66]. Replacing the WB, which can be time consuming and whose interpretation can be subjective, with another serological test, and the use of recombinant or synthetic antigens increased the specificity and sensitivity (at an early stage) of the two-tiered testing [34, 66]. A non-exhaustive list of the different used techniques is reported in Table 2. In this context, Waddell and collaborators reviewed the sensitivity and specificity of these tests according to the stage of the disease and the combination of the approaches [67]. The two-tiered testing is nowadays still necessary because unless single test have some close sensitivity or specificity, none attained or surpassed the traditional two-tiered testing [59].

**Table 2:** Examples of sensitivity and specificity for MTTT in America and in Europe

Methods used		Symptoms / Stage of the disease	Patient's localization					
			**America**			**Europe**		
**First test**	**Second test**		**Sensitivity**	**Specificity**	**References**	**Sensitivity**	**Specificity**	**References**
VlsE C6-ELISA	na	Erythema migrans or early Lyme borreliosis *AP*	29%	96%	[68]	65–68%	91–92%	[34, 61]
		Erythema migrans or early Lyme borreliosis *CP*	56%	96%		80–82%		
		Acute-phase neurologic or cardiac involvement	98–100%	96%		94–100%		
		Arthritis or late neurologic involvement	98–100%	96%		94–100%		
Enzygnost-2®	C6-ELISA	Erythema migrans or early Lyme borreliosis	na			78%	96%	[59]
Whole-Cell Sonicate ELISA	C6-ELISA	Erythema migrans or early Lyme borreliosis *AP*	38–58%	98–100%	[20, 34]	na		
		Erythema migrans or early Lyme borreliosis *CP*	76–79%	98–100%				
Borrelia DotBlot G®	na	nd	93%	35%	[69]			
MarDx® EIA	na		100%	92%				
VIDAS®	na		100%	90%				
VlSE1-IgG	pepC10-IgM	Erythema migrans or early Lyme borreliosis *AP*	46%	96%	[54]			
		Erythema migrans or early Lyme borreliosis *CP*	89%					
		Acute-phase neurologic or cardiac involvement	100%					
		Arthritis or late neurologic involvement	100%					
VlsE1-pepC10	C6-ELISA	Erythema migrans or early Lyme borreliosis *AP*	na			51%	94–95%	[61]
		Erythema migrans or early Lyme borreliosis *CP*				67%		
		Acute-phase neurologic or cardiac involvement				88%		
		Arthritis or late neurologic involvement				90%		

*AP* Acute phase, *CP* Convalescent phase, *na* Not applicable, *nd* not determined

### Antibodies-based approaches

The development of a screening test which would not require specific materials, nor laboratory equipment, but available for a self-test, appears to be an appealing option. One of the main methods with these characteristics, which was widely used for the COVID-19 crisis, is called Lateral Flow ImmunoAssay (LFIA) technology. It has many advantages: it is fast and simple to perform, easy to interpret, and can be performed outside the laboratory. This technology is simple and derived from the ELISA principle. A previously treated and dried sample paper receives the biological sample and the migration buffer. This sample, named analyte, migrates by capillary action on the paper containing a colored particle (latex, colloidal gold…) coupled to Abs against human IgG or IgM, considered as antigen. The analyte-colored particle complex then migrates through a nitrocellulose membrane coated with a bacterial protein able to capture the complex. The formation of the complete complex will provide a colored line according to a sandwich model. A line test containing Ig against conjugated Abs is used for control (Fig. 3B). Few commercialized tests exist, based on the antibody response of the host, such as the Lyme IgM or IgG (VEDA.LAB, Alençon, France) and the Keul-o-test *Borreliose* Complete IgM and IgG test (Borreliose Complete; BioGenTechnologies, Steinfurt, Germany). However, they suffer from low sensitivity (30% on average) despite a quite good specificity: 85% to 88% [70]. The inclusion of a reader, as in the ReaScan + C6 LYME IgG test (index test; Reagena, Toivala, Finland), can help in the analysis of the results to avoid subjective interpretations by the user, which is a major drawback of these assays. For this device, a recent study determined sensitivity and specificity between 83 and 91% respectively [71]. However, these products are currently not recommended for diagnostic use by either the USA or the European institutions, since the sensitivity and specificity are lower than the traditional two-step testing process [72].

Considering the coinfection notion appears highly appealing since ticks transmit several pathogens. In this context, a multiplexed vertical flow assay (xVFA) is under development. It consists of different layers of paper driving a uniform vertical flow of buffer and serum through a detection membrane, allowing the detection of antibodies directed against numerous *Bb*-specific antigens on the sensing membrane such as OspC, BmpA, P41, decorin binding protein B (DbpB), Crasp1, P35, or Erpd/Arp37, as well as the C6-like peptide. A colorimetric signal is observed after 15 min. The specificity and sensitivity of the test are 96.3% and 85.7%, respectively [73].

A new paramagnetic bead-based multiplex assay using Luminex™ xMAP™ INTELLIFLEX System for the simultaneous detection of Borrelia specific IgG/IgM class antibodies allowed to reduce experiment time and biosample material requirements (sensitivity and specificity are reported Table 3) [74].

**Table 3:** Sensitivity and specificity for emerging but not yet accredited laboratory tests

Classification	Tests	Sensitivity	Specificity	References
**Microfluidics,** **lateral and vertical flow immunoassay**	mChip-Ld	84%	92%	[75]
	Lyme IgM and IgG	26%	85%	[70]
	Keul-o-test Borreliose Complete IgM and IgG test	32%	88%	
	ReaScan + C6 LYME IgG	83%	91%	[71]
	Multiplexed vertical flow assay (xVFA)	86%	96%	[73]
**Luminex**	Paramagnetic bead-based multiplex assay and INTELLIFLEX System	94%	97%	[74]
**ELISpot and cells-based approaches**	iSpot LymeTM	54—84%	54 – 94%	[76, 77]
	QuantiFERON-Lyme	70%	na	[78]
	Spirofind	43%	82%	[77]
	LTT-MELISA	30%	53%	
	TCR sequencing	56%	99%	[79]
**Biomarkers detection**	ReaScan CXCL13 assay	78%	95%	[80, 81]
	RecomBead CXCL13 assay	86 – 100%	91 – 97%	
	Quantikine CXCL13 ELISA	88 – 100%	89 – 99%	[81, 82]
	Metabolomics techniques	88%	95%	[83]
**Raman Spectroscopy (RS)**	SERS and DNA aptamers	91%	96%	[84]
	Raman Spectroscopy	85%	90%	[85]
	Raman Spectroscopy & Chemometrics	83%	91%	[86]
**PCR-based approaches**	Immuno-PCR	69%	98%	[87]

*Na* not applicable

Diminishing volumes and reagents is one of the ways to improve the LB diagnosis since the sample collection can be invasive. By combining these characteristics, microfluidics seems the most suitable method. Recently, Nayak et al. developed a point of care test based on this principle. It allows dealing with very small volumes of fluids, down to femtoliters. This rapid 15-min multiplexed laboratory test consists of a chip called mChip-Ld that detects anti-*B. burgdorferi* antibodies using the OspC-K antigen [75]. This test, very similar to LFIA, also uses a signal detection device. Thus, specific antibodies from the patient blood sample will bind to antigens immobilized on the surface of the microfluidic cassette. The peculiarity of this test is the silver amplification protocol to enhance the signal thanks to the silver ion reduction on gold nanoparticles attached to the cassette surface [88]. With a sensitivity and specificity of 84% and 92%, respectively for early diagnosis of the disease [75], this technique is promising, as confirmed by other studies, which showed that it provides an increase in sensitivity compared to the STTT approach [89].

### ELISpot and derived approaches

Many studies show that *Borrelia burgdorferi* can enter endothelial cells and macrophages [90–92]. This ability is one of the main components of the humoral immune escape mechanism of *Bb* [27]. Similarly to viral infections, intracellular activation of type I interferons (IFN) plays an important role in *B. burgdorferi* infection [93]. One of the immune responses of the host in LB infection is characterized by a cytokine response and the secretion of IFN-γ followed by a high expression of IL-4, by T helper 1 lymphocytes, which are associated with non-chronic manifestations of LB. However, persistent IFN-γ expression could lead to chronicity of the LB [94, 95].

In light of this, the secretion of IFN-γ can be exploited to detect the presence of Ag from *Bb* using ELISpot methods [96]. It measures the antigen-specific cellular responses by quantifying the number of IFN-γ-producing T cells [97]. The sensitivity of this kind of method is known to be about 20 to 200 times greater than ELISA or flow cytometry, and it is based on conditions (antigens and cellular medium) used for LT activation [98]. The iSpot Lyme™ is an ELISpot method (Autoimmun Diagnostika / Genome Identification Diagnostics) that uses recombinant *Borrelia* antigens (recombinant DbpA, OspC, p100, and VlsE) to stimulate specific effector/memory T cells combined to a signal enhancer medium CTL Test Plus. This assay showed significantly higher sensitivity (84%) as compared to the WB (30%) [76]. However, as for conventional serology, traditional or improved ELISPOT assays cannot differentiate active from past LB [99].

The ELISpot principle has been combined with the QuantiFERON technology (QIAGEN Sciences) for the detection and measurement of IFN-γ in the case of *Mycobacterium tuberculosis* infection [100]. Taking inspiration from this technology, Callister’s team developed the QuantiFERON-Lyme assay, which consists in the detection of IFN-γ in whole blood after incubation with synthetic *Borrelia* antigens (p66, DbpB, OspC, and flagellin) [78]. This test showed a sensitivity of approximately 70% in patients with EM *versus* 17% for standard serology. In addition, this test allows the distinction between an active or past infection because of the significant decrease of the immune response in patients who have been treated. However, this technique seems to be suitable only in the United States where *Bb*ss, one of the species belonging to the *B. burgdorferi* sensu lato complex, dominates. In Europe, where *B. afzelii* and *B. garinii* are the dominant species, induced responses are low and a more sensitive test is therefore required [101]. The use of INF-γ for diagnosis could be challenging, and dependent on the cell type used for the assay. Indeed, a recent study confirmed that *Borrelia burgdorferi* is a poor inducer of INF-γ production by peripheral blood mononuclear cells (PBMCs) [102] but a strong inducer by human primary NK cells [103]. More studies have to be done regarding specificity and reliability.

Many other methods are based on the ELISpot principle, such as the Spirofind Revised (Oxford Immunotec), which quantifies the IL-1ß produced by primary PBMC cells after contact with *Borrelia* mix, using bead ELISA assay [104].

Also based on T cells, some authors exploited the massive sequencing of T-cell receptor repertoire to highlight the specificity of T-cell responses and probe pathogen exposure. Indeed, it is known that the serological approach based on specific antibody-response suffers from a seronegative window period of 2 to 4 weeks [105], whereas T-cell response is detectable before the humoral response [79]. To circumvent both the co-infection issue and the difference between the kinetics of T-cell response and the humoral response, Greissl and collaborators recently proposed an aid for diagnosis based on a tool allowing to analyze and classify TCR sequencing in order to ensure higher sensitivity testing compared with STTT, in particular concerning early LB (Table 3).

Other tests also based on cellular proliferation are available. A few years ago, a Lymphocyte Transformation Test-Memory Lymphocyte Immunostimulation Assay (LTT-MELISA®, InVitaLab) was developed. This assay is based on two steps: uptake of radioisotope by dividing lymphocytes, followed by their detection [106]. This method evaluates the lymphoproliferative response of PBMCs to *B. burgdorferi* antigens. iSpotLyme™, Spirofind™, and LTT-MELISA® assays have been recently studied and have been compared to classical serology testing [104]. According to the sensitivity and specificity reported in Table 3, it has been confirmed that these cellular tests lead to a high number of false-positive results and are unfit for clinical use at this stage [77].

### Biomarkers-based approaches

The use of the omics (transcriptomics, metabolomics, inflammatomics) approach could identify biomarkers or biosignature of a LB [107]. Several studies have shown that some molecules, related to the immune response of the host, can be used as biomarkers for the diagnosis of LB.

Chemokines and cytokines are key signaling molecules for inflammation and modulation of the immune cells. For Lyme neuroborreliosis (LNB) diagnosis, inflammatory cerebrospinal fluid (CSF) changes (pleocytosis, blood-CSF barrier dysfunction, and intrathecal Ig synthesis) can be expected. Laboratory diagnosis is made by calculating an antibody index based on intrathecal production of antibodies against *Bb* by comparing CSF and serum antibody levels [22, 108, 109]. To improve LNB diagnosis, many studies and tests are based on the quantification of the C-X-C motif chemokine ligand 13 (CXCL13). This chemokine is produced by antigen-presenting cells such as dendritic cells and macrophages. Via its receptor, namely CXCR5, this chemokine is used to guide B cells towards secondary lymphoid organs [110, 111]. Thus, this chemokine has many advantages as compared to the antibody index method, for example (i) its CSF level is high [112, 113], (ii) this chemokine is detectable before antibodies whose levels can be very low at early stage neuroborreliosis [114], and (iii) its level rapidly declines after antibiotics treatment, while CSF pleocytosis remains elevated and antibody level remains positive for years after treatment [115]. Thus, this marker could be an important element for the diagnosis of Lyme neuroborreliosis. Many methods allow its detection and quantification. In a recent study, Haglund and collaborators compared 2 commercial assays: the ReaScan CXCL13 (Reagena Ltd, Toivala, Finland) and the recomBead CXCL13 (Mikrogen Diagnostik, GmbH, Neuried, Germany) assays. The ReaScan CXCL13 assay is a rapid cassette-based immunochromatographic system, similar to LFIA approach. The reader values are translated to semi-quantitative CXCL13 concentrations interpreted as < 250 pg/mL (negative), 250–500 pg/mL (grey zone), and > 500 pg/mL (positive/suspected LNB). As in all LFIA assays, CXCL13 in the sample interacts with an antibody conjugated to colloidal gold, and the complex is then captured onto the test line by anti-CXCL13 antibody. The recomBead CXCL13 (Mikrogen Diagnostik, GmbH, Neuried, Germany) assay, based on the Luminex xMAP® technology, is interpreted as CXCL13 < 190 pg/ mL (negative), 190–300 pg/mL (grey zone), and > 300 pg/mL (positive/suspected LNB). In this type of technology, beads coated with anti-CXCL13 antibodies are incubated with a sample containing unbound biotinylated antibodies against CXCL13. After washing, incubation with a reporter streptavidin-R-Phycoerythrin is made and a sandwich is generated. By using a dual laser system, the presence and intensity of the reporter associated with the bead are detected providing information about CXCL13 concentration in the sample [80]. While the sensitivity of recomBead is higher than that of ReaScan (see Table 3), the recomBead is less specific. ELISA methods can also be used for measuring CXCL13 levels with a sensitivity of 88–100% and a specificity of 89–99% [81, 82, 116]. However, determining levels of CXCL13 as a marker for LNB can aid in the diagnosis but should be interpreted with care since CXCL13 has been reported to be nonspecific to Lyme neuroborreliosis. Increased CSF values have also been found in patients affected by neurosyphilis [117], CNS lymphomas [118] or also in immunocompromised patients and patients with an autoimmune disorder [82]. To overcome this limit, a recent study proposed to titrate the interleukin-6 (IL-6) in addition to the CXCL13 chemokine. High concentrations of IL-6 have been found in CSF samples from patients suffering from neuroinfections due to bacterial or viral etiology, while lower levels have been detected in CSF specimens from cases of LNB. However, the use of CXCL13 and IL-6 needs to be evaluated further in future studies [119].

CXCL9, CXCL10, and C–C Motif Chemokine Ligand 19 (CCL19) levels are significantly elevated in the serum during acute infection depending on the severity of the disease, but they usually decrease after the proper treatment and the resolution of EM [120]. Elevated levels of CXCL9 and CXCL10 are linked to the Th1 immune response due to bacterial and virus infection [121]. The exploitation of these chemokines, knowing that the list isn’t exhaustive and will be enriched in the future, with studies under consideration (such as CCL20, IL-17A…) [122], will therefore give important information for the diagnosis and the understanding of the disease, as well as for the optimization of the treatment, if needed.

Nowadays, a controversy exists when patients, after treatment, develop persistent symptoms, qualified either as chronic LB or post-treatment Lyme disease syndrome (PTLDS). A study showed that CCL19 levels are elevated in patients with the development of these persistent symptoms [123]. The identification of this biomarker, by using a method similar to ELISA and based on the Luminex-xMAP® technology described before, called Bio-Plex bead array system, will offer the opportunity to better understand this phenomenon, its diagnosis, and treatment. Within the related study, 14.5% of treated patients have developed persistent symptoms, and, notably, after 1-year, high CCL-19 levels were only observed in patients with PTLDS, although these observations need to be confirmed by further studies. Other molecules such as autoantigens, specifically apolipoprotein B, present in large quantities in patients with Lyme arthritis, could serve as diagnostic elements [124].

Besides measuring the levels of the above-mentioned chemokines, the analysis of metabolomics can be used to determine molecules exploitable as biomarkers or biosignatures of specific disease states [125]. Indeed, since metabolic activity strongly depends on environmental factors, including infections [126], the liquid chromatography-mass spectrometry approach could be potentially exploited for diseases diagnosis, as it has been shown for the diagnosis of schistosomiasis [127]. This approach seems to be promising for the LB diagnosis as demonstrated by Molins and collaborators who established a metabolic signature with a sensitivity of 88% and a specificity of 95% for the early detection of LB [83]. Using a similar method, some isoprostenes and neuroprostane, such as malondialdehyde and 4-hydroxy-2-nonenal, can serve as biological markers [128]. Indeed, Ligor et al*.* showed that the identification by spectrometry and liquid and gas chromatography of these compounds in urine, blood, or CSF can help to diagnose the early stage of Lyme disease. Another recent and interesting research article from Magni et al*.* aims to present a new experimental procedure for characterizing the urinary pathogen-derived proteome of patients from LB. This innovative method, combining the mass spectrometry technique with a sample concentration methodology (affinity hydrogel particles) and a bioinformatic authentication method, allows to better identify patients with the presence or persistence of the *B*LG, even after antibiotic treatment [129].

Also based on biosignatures, many interesting research articles investigate the potential Raman spectroscopy (RS) diagnostic capacity for LB. This method does not detect *Bb* spirochetes in blood, but rather biochemical changes associated with *Bb* infection. Goff et al. demonstrated that blood samples from mice but also from humans were analysed with quite high sensitivity and specificity [85, 130]. In a similar approach, combining RS with chemometrics, Senger et al. developed an interesting test allowing to distinguish an LB molecular signature from healthy volunteers, end-stage kidney disease patients, and patients with active or remissive bladder cancer (Table 3) [86].

Altered metabolic biomarkers profiles can reflect a disease state and be exploited for LB diagnosis. In this context, and by analyzing the metabolic pathways of *Borrelia* bacteria, it has been shown an evolutionarily reduced genome because of its close association with its vertebrate and tick hosts [131]. For this reason, many metabolic pathways lack making the bacteria incorporating host lipids for growth. By including host’s lipoproteins and lipids, such as phosphatidylcholine (PC), phosphatidylglycerol (PG), phosphatidylserine (PS) and cholesterol, the host immune system develops autoantibodies to those lipids. The detection of the elevation of antiphospholipid antibodies against PC, PG, or PS, but not against cardiolipin, may first aid in the diagnosis of Lyme disease, but also distinguished LB from syphilis and some other diseases [132, 133]. Interestingly, Molins et al.managed, by analyzing a metabolomic signature, to differentiate early LB from southern tick − associated rash illness (STARI) with an accuracy of 85 to 98%. Indeed, early LB can be clinically diagnosed by EM, but can be confused with other illnesses like cutaneous manifestation from STARI [134].

In combination with AI approach, some studies linked to biomarkers are reported in the Sect. 4 [135–137]. These innovations pave the way for an approved diagnostic test.

## Direct diagnosis

Direct diagnosis, mainly based on the detection of the pathogen, is for many infectious diseases the golden standard for proving an active infection. Currently, a direct diagnosis of a disease can rely on many methods: culture of the pathogen, microscopic observation, xenodiagnosis, detection and amplification of the pathogen DNA/RNA, and proteins/antigens (Ag) detection [36].

### Culture of *Borrelia burgdorferi*

The culture of *Bb* represents a difficult task at multiple levels. First of all, obtaining samples can be extremely invasive. Indeed, although blood or urine samples can sometimes be used to collect the etiological pathogen of LB, in some cases skin biopsy from EM or ACA, or even synovial, cerebrospinal fluid (CSF) or myocardium need to be used [138, 139]. Therefore, the patient’s pain, the associated risks, the available technical skills, and the relative costs need to be considered when culturing *Bb.*

In addition, the *Borrelia* culture is a fastidious and long process, making this approach unsuitable for rapid detection of the pathogen. Indeed, *Bb* grows on a very specific and rich medium, i.e. Barbour-Stoenner-Kelly (BSK) – II or BSK-H supplemented with rabbit serum or modified Kelly–Pettenkofer (MKP) [140]. Many comparisons have been done regarding the interest of each medium. For example, according to a study from Ružić-Sabljić et al., BSK-H medium supports the growth of borrelial strains but MKP is superior with regard to the isolation rate, morphology and motility of strains. However, even if BSK-H medium supports fast initial growth of *borreliae*, this is followed by rapid deformation and death of the spirochaetes [141]. Regarding the different genospecies insides *B*LG, the comparison of MKP and BSK-H medium for *Borrelia* culturing from skin specimens of European patients with EM revealed the advantage of MKP over BSK-H [142]. Indeed, when using *Borrelia* isolates from tick- or host-derived samples, MKP medium should be preferred [143].

The growth rate ranges between 8 and 12 h at the appropriate temperature (between 30 and 35 °C) [144], which justifies that pathogen detection takes around 24 days for blood culture and skin biopsy samples [145]. Furthermore, the use of a rich medium makes the culture more sensitive to contamination by fast-growing bacteria. Thus, to prevent bacterial contamination, an antibiotic should be used, such as rifampicin or phosphomycin, since *Borrelia* is intrinsically resistant to these antibiotics. Amphotericin B, in combination with rifampicin and phosphomycin, can also be used to suppress fungal growth [146]. Therefore, the culture should be kept for at least 8 to 12 weeks to consider a negative result [29]. As previously mentioned, despite *Bb* culture being considered as the gold standard with a specificity close to 100%, the sensitivity of this approach remains very low, depending on the clinical stage of the disease, the sample origin, and the genospecies responsible for LB [147]. Sensitivities as a function of the type of clinical sample are reported in Table 4. Moreover, the clinical samples often contain a small number of living bacteria, sometimes lower than the measurable level, and the authors underlined the extreme variability of borrelia quantity collection in samples [36, 148, 149]. Finally, previous works suggest that *Bb*sl could adopt a persistence state including a viable-but-nonculturable (VBNC) state [24, 150]. This, together with the above-mentioned sample harvesting and bacterial growth problems, puts bacterial culture beyond the capabilities of most clinical laboratories and therefore not suitable for diagnosis.

**Table 4:** Sensitivity of culture detection and PCR assays depending of the origin of clinical sample

Methods	Origin of clinical sample	Sensitivity range	References
**Culture of *B. burgdorferi***	Skin biopsies from EM	40 – 90%	[36, 149, 151]
	Skin biopsies from ACA	20 – 60%	[36]
	Skin biopsies from Borrelial lymphocytoma	24 – 32%	[147]
	Synovial fluid/biopsy	< 1%	[36]
	Cerebrospinal fluid	10 – 26%	[29, 36]
	Blood	5 – 40%	[29, 36, 149]
	Urine	nd	[152]
**Traditional PCR assays**	Skin biopsies from EM	30 – 89%	[29, 153]
	Skin biopsies from ACA	20 – 100%	
	Skin biopsies from Borrelial lymphocytoma	67.5%	[154]
	Synovial fluid/biopsy	40 – 96%	[36, 155, 156]
	Cerebrospinal fluid	5 – 30%	[36, 147, 155]
	Blood	10 – 20%	
	Urine	nd	[152]

*Nd* not detectable

### Microscopic observation and xenodiagnosis

The clinical utility of direct microscopic observation of *Bb* is limited due to the low concentration of bacteria in body fluids. Indeed, Wormser et al. considered the number of 0.1 cultivable cell/mL of whole blood [149], even if such quantification can vary according to the authors [148, 157].

This low load means that the detection by light microscopy is not feasible in clinical practice, ruling out such an approach for routine diagnosis. Furthermore, the specificity of this approach is moderate, as artifacts can indeed be responsible for false positive interpretations, even by a trained biologist. For example, recently, Laane et al. carried out a study based on a modified dark-field microscopy technique, allowing the detection of *Borrelia* structure [158]. However, using the same method, Aase et al. showed that 85% of blood samples from the healthy control group were detected positive when only 66% of those with LB were detected, making this approach unsuitable for Lyme disease diagnosis [159].

Alternatively, the presence of an infection can be determined by xenodiagnosis. This method is based on the biting and feeding of an uninfected tick on the suspected infected patient. Then, the tick is used to check the presence of *Bb* by Polymerase Chain Reaction (PCR) assay. Trials have been performed in murine models or in Rhesus Macaques [160, 161]. In this last-mentioned trial, small numbers of intact spirochetes were recovered by xenodiagnosis from treated monkeys, also demonstrating that *B. burgdorferi* can withstand antibiotic treatment. Even if xenodiagnosis appears safe and generally well tolerated in humans [162], Bockenstedt and Radolf underlined a flawed result since xenodiagnosis works in inbred mice because spirochetes persist in the distal skin, but not in humans [163].

### Antigens detection

Antigen detection assays suffer from the same limitations as microscopic detection. Few diagnostic tests capturing antigen from *Bb* exist [164–166] and the reliability of these methods for clinical practice is poor [36]. Due to low level of bacteria in body fluids, which depends mainly on the origin of the sample or the LB stages, many methods have been developped to improve antigen/bacteria detection in a clinical sample. They consist in, but are not restricted to, the concentration of antigens or bacteria, increasing of the initial clinical sample volume, and improvement of antibodies affinity, peptides, or aptamers for one or several specific targets.

With this aim, centrifugation helps to concentrate membrane proteins (OspA, flab, p66, OspC and BmpA) after bacterial lysis. This method allows the detection of *B. burgdorferi* membrane OspA proteins at low concentrations of about 4 fmol OspA/mg serum protein [167]. Other concentration methods such as the Nanotrap [168] or the Hydrogel Microparticles technology [169, 170] are also available. In both approaches, nanoparticles will bind to the OspA antigen in urine or any other body fluid. An elution can then be performed in a smaller volume allowing its concentration before detection using any immunoassay technique. Magnetic particles as similar diagnostic tools can also offer a large signal amplification by concentration of Lyme antigens or by bonding many labels such as enzymes (like horseradish peroxidase (HRP)) [171].

Regarding the affinity of molecules to their target, a recent study presents an improved direct method, where the protein OspA is detected in clinical serum samples, combining enhanced Raman scattering (SERS) and aptamers. A DNA aptamer, exhibiting a high specificity and a dissociation constant (K_D_) of 2.18 nM for OspA, shows a sensitivity of 91%, using serum samples from Lyme patients. In addition, the OspA limit-of-detection was determined to be 1 × 10^−4^ ng/mL, four orders of magnitude lower than that found in serum samples from early Lyme disease patients. This promising application may help Lyme diagnosis [84].

Flynn et al. utilized the inherent binding affinity of the BBK32 protein for human fibronectin to propose a highly promising electrochemical sensing methodology. As a proof of concept, they employed a biomimetic electrode to measure impedance changes induced by protein interactions, thereby detecting the presence of bacteria [172].

### Traditional PCR assays and PCR improvements

PCR is a widely known in vitro method for the amplification and detection of DNA. Briefly, copies of a targeted DNA fragments will be recovered, if present, in the sample. *B. burgdorferi*’s genome which is very complex and consists of a ~ 950-kb linear chromosome and many additional circular plasmids and linear plasmids that range in size from 9 to 62 kb [173]. Because of this complexity, many genes can be chosen as the target DNA fragment, like a unique rRNA gene (*16S rDNA, 23S rDNA, 23S-5S rDNA* intergenic spacer), or a single gene in the *Bb* chromosome (*flagellin (fla), hbb, rrf-rrl, polC, SrRNK, p66, recA, bmpA, rpoB, rpoC, or gyr*). Regarding the plasmid targets, many genes can be used such as *dbpA, vlsE,* and outer surface protein genes (*ospA, ospB, ospC*) [155]. The sensitivity varies depending on the sample. Interestingly, authors recently demonstrated significance to perform an early PCR detection of *Borrelia,* directly on the bite site, using a new non-invasive sampling device, based on a microneedles patch [174].

Table 4 reports the sensitivity of PCR assays according to the origin of the clinical sample. As before mentioned, besides the very low level of bacteria according to the sample collections, the main limitation of PCR assay is its inability to distinguish between active and past infection, since *B. burgdorferi* DNA remains present for weeks, or even months, after antibiotic treatment [11, 175]. Additional reasons justify this limitation: Firstly, nondividing *Borrelia* can remain in body fluids and can be detected, leading to positive results despite past infection [176, 177]. Moreover, it has also been shown that *Bb* produces outer membrane vesicles containing virulence factors, such as Outer surface proteins (OspA, B, and C), as well as DNA [178]. Finally, even if antibiotic treatment has been successful, the residual DNA or antigenic proteins included in such blebs could induce positive results despite a past infection.

Thus, no conclusion can be made by PCR on the discrimination between residual DNA and viable organisms, or the success or lack of, a therapy.

As previously mentioned, the improvement of traditional PCR assays is one of the top research subjects for LB diagnosis. Many studies showed that a simple way of improving the sensibility of PCR assays can rely on the preparation and enrichment of bacteria/DNA in the clinical sample. For example, the application of a centrifugation step to concentrate the bacteria in the sample, the use of cDNA as a template, and the removal of erythrocytes improve the sensitivity of PCR assays [179]. Alternatively, a lysis step of PBMCs containing masked intracellular bacteria can be added, increasing the amount of *Bb* DNA copies in the initial sample. For this purpose, different lysis techniques can be used such as hypotonic water shock, solutions containing ammonium chloride (NH_4_Cl), or the use of chaotropic and/or detergent agents (e.g. SDS, triton, etc.) [180].

**Fig. 4:**
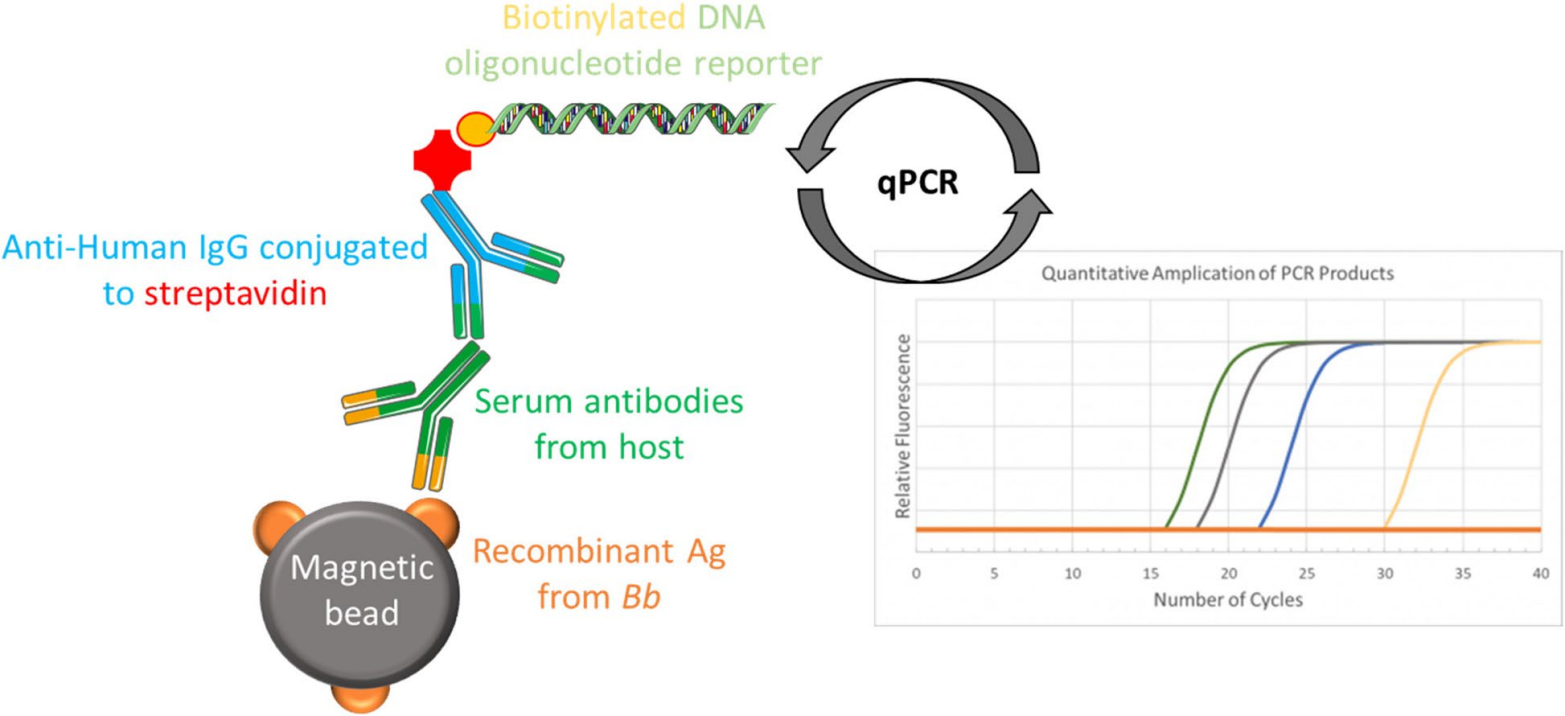
iPCR principle

An innovative method based on PCR called immuno-PCR (iPCR) allows to increase the detection limit, for some cases, of a conventional ELISA about 100- to 10,000-fold [181, 182]. Applied to Lyme borreliosis diagnosis, the immuno-PCR uses a recombinant *B. burgdorferi* protein antigen coupled to magnetic beads to capture *B. burgdorferi*-specific host-generated antibodies. A biotinylated DNA oligonucleotide reporter molecule coupled to a streptavidin-conjugated reporter antibody is then amplified by qPCR for detection and quantification (Fig. 4). This method, described by Halpern et al., shows better sensitivity (reported Table 3) than the STTT method. [87, 183].

Besides these considerations, the PCR approach should also consider potential co-infections. Indeed, cases of co-infections comprising two or more species belonging to the genus *Borrelia,* but also cases comprising the genus *Borrelia* and other pathogens such as *Rickettsia* spp or *Babesia* spp are reported: According to Raileanu et al., 64.5% of infected *I. ricinus* were positive for more than one pathogen. In Romania for example, the most common co-infection was between *B. garinii* and *B. afzelii* (4.3%), followed by *B. garinii* and *B. lusitaniae* (3.0%). Co-infections between *Borrelia* spp. and *Rickettsia* spp. represented 1.3% of the investigated cases [31]. In addition, cases of human co-transmissions and co-infections have been listed [33, 184–187]. Using traditional PCR assays, the identification of the different species within the complex sensu lato can be based on a multi-locus detection [188, 189] ensuring the differentiation of *Bb* strains. At this scope, multi-locus sequence typing (MLST) and variable number tandem repeat (VNTR) analysis are commonly used and are based on housekeeping genes such as the intergenic spacer rrs-rrlA (IGS) and the ospC gene [190, 191]. Indeed, these sequences allow the distinction between strains because they represent different alleles.

These co-infections change the severity of the symptoms and the effectiveness of the treatments. This shows the need for a multiplexed test on several strains of the *Borrelia* genus, but ultimately for a multiplexed test against all tick-borne diseases. In this context, much effort still must be made to develop a multiplexed PCR to increase the attractivity of this method for clinical use and to assure proper treatment. Another type of PCR, called quantitative PCR (real-time PCR or qPCR) has shown to be able not only to quantify the DNA and to determine the amplification purity by analysis of the melting curve, but also in an original manner, it has been used to detect different species namely *B. burgdorferi, B. afzelii*, and *B. garinii* by using a post-PCR denaturation profile analysis and a single molecular beacon probe. This latter is a hairpin-shaped oligonucleotide probe, highly specific for its target sequences, and usually labelled with distinguishably colored fluorophores [28]. Another qPCR method is based on FRET (Fluorescence Resonance Energy Transfert) and allows to improve the detection limit, with only 10 copies of *Borrelia* per PCR reaction [192]. Thus, the development of these types of multiplexed PCR methods allows for the identification of multiple tick-borne pathogens such as *Bb*sl, *Anaplasma/Ehrlichia* spp., and *Babesia* spp. [28, 193, 194] helping the future diagnosis and choosing the needed treatment.

**Fig. 5:**
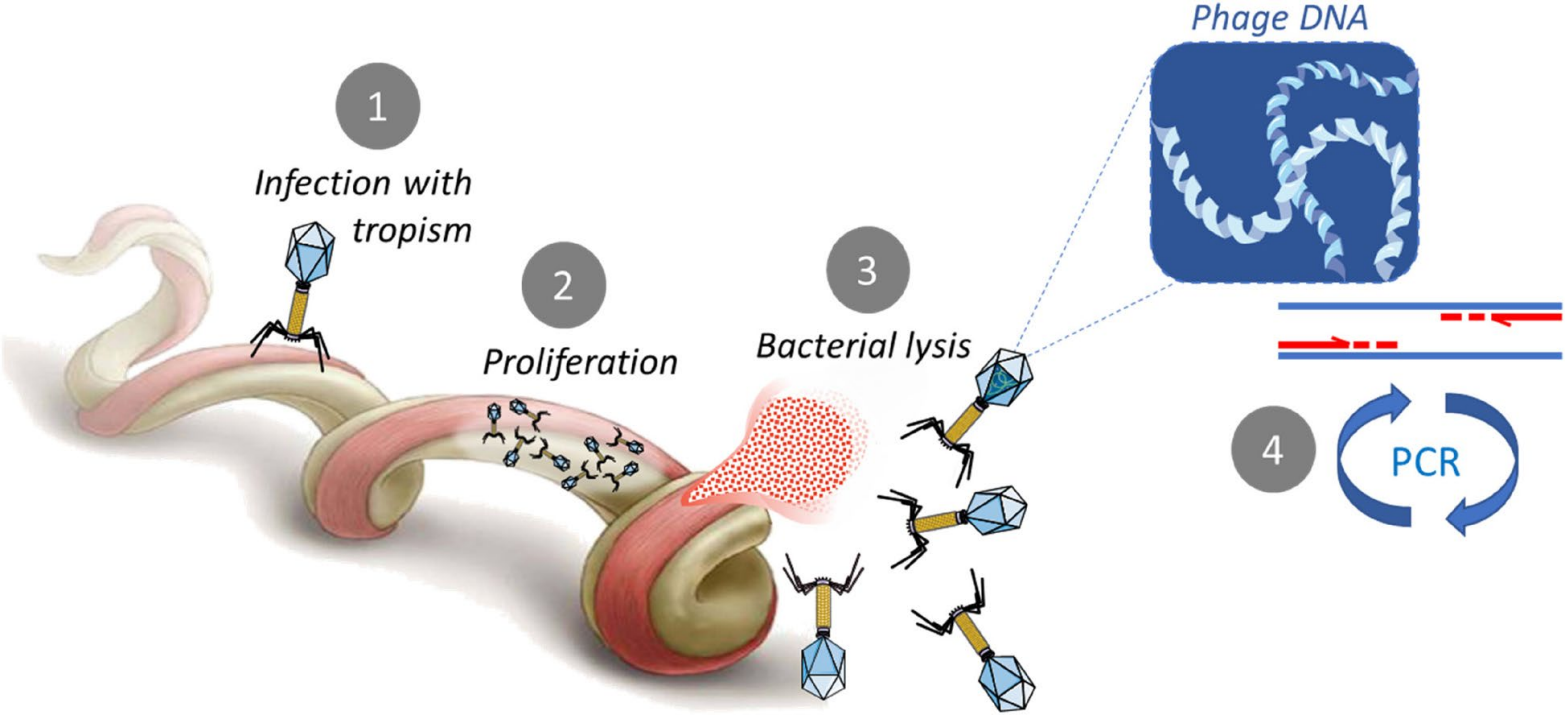
Phage PCR principle

As previously mentioned, one of the major limits of classical PCR assays is the difficulty in distinguishing between living and dead organisms, and therefore between an active or past infection [175]. A new method exploiting bacteriophages has been recently developed to face this issue. Bacteriophages, viruses which infect bacteria, are only present in active bacterial infections since they require living bacteria for their replication. Therefore, a phage-based test is considered as direct evidence of an active infection. An important characteristic of bacteriophages is their ability to shift from a state of lysogenic activity to a lytic one following unfavorable physicochemical and/or biological conditions. Thus, the lytic stage will lead to the release of phages that had proliferated -and therefore of viral DNA- using the host's cellular machinery, and finally lead to the lysis of bacteria [195] (Fig. 5). The high number of viral DNA copies determines and increases the test sensitivity since the presence of one copy of the bacterial DNA implies the presence of many viral DNA copies. Moreover, bacteriophages have a specific tropism towards certain bacteria. In other words, phages infect a specific bacterial species and their genetic material is specific to the bacteria they infect, resulting in a high specificity of the test exploiting this approach, such as the Phelix Phage test patented by Dr. Louis Teulières [196].

The applied principle is the following: after the extraction of the pathogenic DNA from blood samples, a PCR for the detection of phages is performed using primers known to specifically target distinct phages [157]. The identification of the primers panel allowing amplification leads to the identification of the bacteria panel having infected the patient. Finally, the amplified fragments are sequenced to confirm the positivity of the sample and exclude false positives. Based on this principle, a recent study managed to reach a sensitivity of 3.3 *Borrelia* cells per ml of blood, which corresponds to the bacteria level in LB in clinical blood samples, by targeting the prophage *terL* gene (NC_000948.1) [157]. This gene encodes for the terL, a protein responsible for packing phage genomes that were found in three linear plasmids and seven of the circular plasmids of the cp32 series. These latter have been determined to be *Bb* prophages within the B31 genome [197, 198]. Even if this technique is promising, an analysis of this study shows inaccuracies in terms of statistical analysis or cohort composition and comparison with healthy controls, thus requiring validation [199].

## Artificial Intelligence (AI) advent in diagnosis

When it comes to disease diagnosis, accuracy is critical to avoid therapeutic wandering and to plan and prescribe an effective treatment, ensuring the well-being of patients. Artificial Intelligence (AI) can be a strong ally in this scope, as shown by [200]. It could be exploited for LB diagnosis, for example to identify new biomarkers of the pathology. However, it must be recognized that AI studies are still in progress. Their validation must be realized and confirmed. In this context, a machine-learning algorithm was developed and it allowed identification of 20 genes that discriminated LB from other bacterial and viral infections [135]. Interestingly, these novel LB biomarkers not only identified subjects with acute disseminated LB, but also distinguished between subjects with an acute and resolved disease with 97% accuracy. Alternatively, the machine learning-based analyses of RNA-Seq data are promising and allow Servellita et al. to define 31 differentially expressed genes in PBMC cells between healthy and LB patients (Table 5) [136]. Similarly, an up-to-date research article demonstrates that machine learning has managed to identify 35 genes as biomarkers for post-treatment LB. A RNA sequencing of PBMCs patients with post-treatment Lyme disease showed a differential expression with acute LB patients and uninfected people [137].

**Table 5:** Sensitivity and specificity of tests relied on AI

Classification	Tests	Sensitivity	Specificity	References
**Bioinformatics approaches**	Transcriptomic: 20 classifier genes as biomarkers	100%	90%	[135]
	Transcriptomic: 31 differentially expressed genes in PBMC cells	90%	100%	[136]

In a near future, AI tools will reach new heights, and increase the accuracy and velocity of LB clinical diagnosis. For example, studies showed that deep learning, based on a dataset of medical images, which allows the discrimination of EM from ambiguous skin manifestations such as cellulitis or herpes zoster (which can be simulated by LD [201]), can help clinicians with early diagnosis and reduce further complications [202, 203].

## Conclusion

Lyme disease is the most frequent tick-borne disease in the world. Even if EM is the traditional clinical manifestation, the pathogen can spread to other tissues and organs, leading to blurred symptoms. Many direct and indirect tests for Lyme diagnosis are available. Despite the innovation of new or improved assays, the diagnosis of LB remains a challenge. Immune escape mechanisms, the difference in protein and antigen expression, and the low level of bacteria in clinical samples are some of the factors that explain the difficulties encountered. Due to the heterogeneous distribution of genospecies in the world, unique recommendation, unique target and unique technology do not appear to be appropriate for the diagnosis of LB. Not only sensitive and specific assays but also multiplexed tests are required, in order to identify the co-infectious profile of patients frequently mentioned in such pathologies so as to adapt the appropriate treatment. Therefore, it appears that more work needs to be done to develop reliable and unambiguous diagnostic test for LB. Moreover, clinicians need assistance with diagnostic evaluation since symptoms are diverse and blurred. In such a context, the development and application of AI techniques could improve detection and diagnosis of LB.

## Data Availability

Not applicable.
